# Occupants’ Perceptions of Amenity and Efficiency for Verification of Spatial Design Adequacy

**DOI:** 10.3390/ijerph13010128

**Published:** 2016-01-14

**Authors:** Sangwon Lee, Kwangyun Wohn

**Affiliations:** Graduate School of Culture Technology, Korea Advanced Institute of Science and Technology, Daejeon 34141, Korea; wohn@kaist.ac.kr

**Keywords:** spatial design adequacy, future occupants’ perception, satisfaction, amenity, efficiency, perception processing elaboration

## Abstract

The best spatial design condition to satisfy the occupancy needs of amenity and efficiency is determined through analyzing the spatial design adequacy (SDA). In this study, the relationship between the space design elements and space on future occupants’ perception are analyzed. The thirty-three participants reported their self-evaluated SDA that describes the quality of eight alternative housing living rooms with different spatial factors. The occupants were guided through the perception processing elaboration in order for them to evaluate the actual perception in the real space. The findings demonstrated that the spatial size (e.g., width, depth, and height) is significantly correlated with the overall satisfaction of amenity. It is also found that the spatial shape (e.g., the width-to-depth ratio, the height-to-area ratio, and room shape) may significantly influence the overall satisfaction of efficiency. The findings also demonstrate that the causal relationship between the spatial factors and space is clearly present in the occupants’ perception, reflecting the time-sequential characteristics of the actual experience divided into amenity and efficiency. This result indicates that the correlation between the spatial factors and space of SDA under the occupants’ perception processing elaboration can be a useful guide to predict the occupancy satisfaction of amenity and efficiency in real spaces.

## 1. Introduction

### 1.1. Problem Statements and Objectives

In the occupants’ self-reported verification process of spatial design adequacy (SDA) to satisfy their needs in real spaces, the examination of the relationship between the spatial factors and space is essential in order to determine the best spatial conditions [1,2,3,4,5,6,7,8]. For the SDA of safety, health, and sustainability, the occupancy needs are objectively verified because these types of occupancy can be simulated using previous empirical data from real spaces [9,10,11,12,13,14,15]. However, the SDA of amenity and efficiency has not been clearly verified, because the occupants could not clearly state whether the space can satisfactorily support their life considering their social, cultural, and personal backgrounds as well as their experiences [16,17,18,19,20,21]. Due to these problems, the dissatisfaction of amenity and efficiency and the resulting economic loss that occurs after the completion of construction have rarely diminished in real spaces [22].

Spatial design adequacy (SDA) refers to the spatial design qualities that are able to satisfactorily obtain an occupancy goal (in particular, the amenity and efficiency values) in a space. Amenity (comfort) refers to the goal that occupants feel pleasantness in agreeable spatial conditions that result from agreement with their lifestyle and social, cultural, and personal backgrounds as well as experiences [23]. Efficiency (convenience) refers to the goal that occupants do not feel stress in agreeable spatial conditions that results from engaging in certain specific behaviors [24]. Space is generally determined through the combination of spatial factors such as width, depth, and height as well as their respective ratios. They are united with complementary relationships. Therefore, both spatial factors and space, as well as their relationship, should be analyzed in order to determine the best design through the verification of the SDA.

In previous studies, the amenity and efficiency SDAs were reviewed through comparing spatial designs with post occupancy evaluation (POE) results of the referenced actual space [25,26]. However, this comparative evaluation using the referenced POE data has the inevitable limitation of clearly verifying the design quality and whether it satisfies the future occupants’ needs. Also, the relationship between the spatial environment factors and space was studied in green building terms using several rating systems [27,28,29,30,31]. The change of spatial factors such as width, depth and ratio of window to wall influences occupants’ perception of space, because the change affects indoor environmental factors which affect human comfort. However, the influence of spatial factors on occupants’ perception of amenity and efficiency has not been fully discussed, since the effect of environmental factors on occupants in space was limited to certain interactions between them.

A virtual reality environment (VRE) was used to evaluate the SDA for amenity and efficiency, because it exhibits the strength to compose the predictive future occupants’ living scenes in the virtual environments, although VRE has some limitations to predict all of actual experience in real-world environments [32,33,34,35,36]. Various predictive evaluations of SDA using the VRE have been conducted through analyzing the future occupants’ self-reported satisfaction in order to satisfy their amenity and efficiency needs [33,37,38,39,40,41].

However, the occupants’ self-evaluative responses in the previous studies did not clearly describe the SDA to ensure the best housing choice in the actual space. Several shortcomings in the occupants’ responses were found, such as the relationship had not been clearly represented regarding what spatial factors influence the space [19,42,43,44,45]; the actuality was not effectively investigated regarding whether the occupants’ response reflected the characteristic of the actual experience in the real space or not [37,46,47,48,49,50,51]; and redundancies between amenity and efficiency were found regarding whether the occupants distinguished their response in the same spatial conditions or not. These ambiguities in the occupants’ responses have appeared frequently in previous research, as well as in the POE. Due to these ambiguities, the occupants’ predictions in their self-reported-responses have not been objectivity accepted when selecting the best spatial design conditions in the verification process of SDA.

The cause of the ambiguities in the previous studies can be found in the occupants’ perception process [52]. That is, insufficient activation of the occupants’ perception processing [46,47,49] resulted in unclear examinations of the SDA through the self-evaluation process [23,24,53,54,55,56,57,58]. For example, misinformation effects prevented the occupants’ perception from focusing on precise comparisons with their actual experience in order to satisfy their amenity and efficiency needs [59,60,61,62,63]. Furthermore, the evaluation procedure was not guided as a structured procedure in order to clearly describe the relationship between the spatial factors and the space [46,64,65,66,67,68,69]. In human-environment-interaction studies, it is conceived that the occupants’ evaluative perception could be sufficiently elaborated in order to present the causal relationship between the spatial factors and space through a psychological operation, such as a reinforcement of intentional system [48,70,71,72] as well as the improvement of the evaluative participatory procedure in the VRE [4,55,62,73,74,75,76,77].

Therefore, the evaluative perception processing elaboration through the occupants’ evaluation process should be induced in order to predict their satisfaction at a conscious level as well as at the subconscious. The objective of this study is to determine the spatial design conditions under virtual reality in order to satisfy the occupancy needs of amenity and efficiency based on occupants’ evaluations on SDA. In particular, the causal relationship between the space design factors and space under perception processing elaboration was discussed to activate the actual predictive occupancy satisfaction evaluation.

The thirty-three participants evaluated the quality of the eight alternative housing living rooms with different spatial factors constructed in the VRE. The perception of the spatial factors (*i.e.*, spatial size, shape, and configuration) and overall satisfaction of space were examined through the self-evaluated reports, and their relationships were analyzed. The influence of indoor environmental factors on occupants’ perception of amenity and efficiency was not discussed since the tests were performed based on VRE.

### 1.2. Related Theories and Analysis Framework

Occupancy satisfaction evaluation could be described as a decision-making process to select a certain expected spatial values that could lead to effective results in association with occupants’ subjective views. This study focuses on evaluation of an activated new experience (prospective memory) on current spatial design compared with their previous experience of amenity and efficiency (retrieved memory) under perception processing elaboration. Various relevant theories are applied to establish hypotheses and analysis results of this study. 

Expectancy-value model and the theory of planned behavior as well as decision-making approach were reviewed to establish hypotheses and experimental conditions. Housing preference and choice has been studied from different theoretical perspectives [74].

Human-environment-interaction theories are applied to determine a characteristic of actual experience in analysis of participants’ responses. Experiential aspect of amenity and efficiency on occupants’ are analyzed in the respect of pleasure-arousal hypothesis and social learning theory [52]. Concepts of affordance and satisfaction are used to describe the structure of the causal relationship between the space design factors and space in occupants’ perception [18,78,79,80].

Cognitive psychological perspective is used to observe the distinguished aspect between amenity and efficiency in correlations and to control the experimental conditions. This study investigates perceptions in time-sequential under distinguish between perception (instant evaluation for spatial element in immediate) and cognition (holistic evaluation for space by recall) [52]. Priming is adopted to elaborate occupants’ evaluative perception processing during the evaluation, *i.e.*, introduction of categorical knowledge for amenity and efficiency and pre-accommodation in VRE. This would affect to integrate top down perception between bottom up perception with the intentionality for amenity and efficiency in association with time-sequential causality [80].

Neuroscience researches are also considered to analyze the perception analysis. In this study, the occupants’ perceptions could be divided according to their activated levels of perception. Amenity could be an emotional reaction to the emotional background attention, and it requires involuntary withdrawing of the implicit memory. In contrast, efficiency could be an attitude toward certain actions in the goal-oriented attention, and it requires voluntary intensive withdrawing of the explicit memory, such as categorical knowledge [64,65,66]. In this study, amenity was observed as a perception of free acts compared with previous experiences in the real space. Efficiency in the study was observed in predicting the movement for serving guests. Efficiency requires more attention than amenity in the perception processing elaboration.

### 1.3. Hypotheses

A series of hypotheses were established in order to verify the causal relationship regarding the occupants’ perception in association with their actual experience. Three hypothesis groups were proposed under the concept of human-environment-interaction theories as well as consideration of the previous studies, as follows.

- *Hypothesis group 1 demonstrates that the spatial size factors such as width, depth, and height of the space affect the perception of amenity and efficiency.* Most previous studies have not clearly described the spatial conditions required in order to satisfy occupants’ amenity and efficiency needs regarding the relationship between the spatial factors and space [35,81,82,83]; Fang described that the unit size and the length of stay were significantly related to residential satisfaction [84]. In human-environment-interaction theory, the pleasure-arousal hypothesis demonstrates that occupants were most attracted to settings that were moderately arousing and maximally pleasurable [52]. Occupants feel pleasantness in agreeable real spatial conditions resulting from agreement with their openness experiences, e.g., a high height and wide width of the space.
- *Hypothesis group 2 demonstrates that the spatial shape factors such as the width-to-depth ratio, the height-to-area ratio, and the room shape affect the perception of amenity and efficiency.* Most previous studies have not clearly described the spatial conditions required in order to satisfy occupants’ amenity and efficiency needs regarding relationship between the spatial factors and space [35,81,82,83]; Frontczak reported that the amount of space regarding the workspace contributes significantly to the overall satisfaction among the 15 available parameters [16]. In human-environment-interaction theory and social learning theory, occupants observed, processed, and imitated the behaviors, attitudes, and emotional reactions for desirable behaviors [52]. Occupants do not feel stress in agreeable spatial conditions that results from engaging in a certain behavior, e.g., serving guests in a living room.
- *Hypothesis group 3 demonstrates that the overall satisfaction of amenity and efficiency is perceived differently in the same space.* Occupants’ overall satisfaction of the space will be distinguished between amenity and efficiency. Amenity satisfaction is influenced by the spatial aspects (e.g., ceiling height) that affect the emotional satisfaction; otherwise, the efficiency satisfaction is affected by the spatial aspects, e.g., distance to the outdoor area because it affects the movement for hosting guests.

## 2. Methods

### 2.1. Space Selection and Spatial Factors

**Figure 1:**
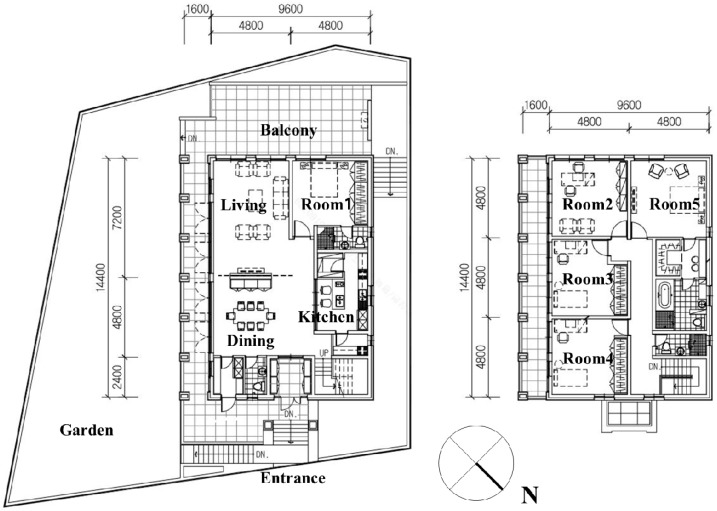
House plans (**Left**: 1st floor, **Right**: 2nd floor).

A house was selected for use in examining SDA. The layout of a typical two-story structure with a total floor area of 260 m^2^ is presented in Figure 1. An important consideration for the future occupants’ satisfaction is to secure the maximum view from the living room that faces the southwest direction. In addition, a balcony should be attached to the living room so that the indoor space is directly connected to the outdoor surroundings. Therefore, the living room was selected for the focus of the experiments because it enabled the investigations of various aspects of amenity and efficiency.

The spatial factors of width, depth, and height were primarily examined. In general practice, in the schematic design phase, the designer and client determine the shape of the space through the selecting best alternative for spatial factors arrangement, e.g., height, width, depth, *etc.* All spatial factors of alternatives were manipulated within the limitations, *i.e.*, budget, building codes, *etc.* For example, in this study the height of living room was limited to 2.8 m which is regulated by the architectural code. The floor area of the space is mainly considered and compared with the future occupants’ requirements during the pre-design phase.

**Figure 2:**
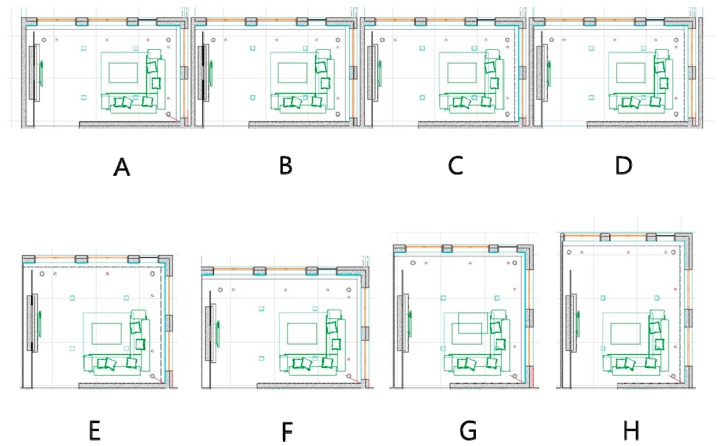
Layout of the spaces created virtually for the living room (Ceiling height varied for **A**–**D**, Base scenarios = **A**: 2.4 m; Room shape varied for **E**–**F**, Base scenarios = **G**: 6 m × 6 m).

A total of eight alternatives were prepared according to the height changing (four alternatives) and layout changes (four alternatives). The height changes in the first group were suggested in order to select the best condition of height and the floor shape changes in the second group were presented in order to select the best condition of floor layout. The area and volume of each alternative was equal. Non-fixtures such as furniture had limited changes. Material and color of finishes were not changed. In this study, the variations of finish material and color were not the influential factors that affected the spatial perceptions. Eight living room spaces were designed according to Korean national building codes. The detailed dimensions of the spaces are summarized in Figure 2 and Table 1.

**Table 1:** Living room dimensions.

Category	Space	Width (m)	Depth (m)	Height (m)	Area (m^2^)	Finishes
I	A (base scenario)	7.33	4.55	2.4	33.35	- Floor :brown wood - Wall : light grey paper and wood - Ceiling : medium gray paper
	B	7.33	4.55	2.8	33.35	
	C	7.33	4.55	2.5	33.35	
	D	7.33	4.55	2.6	33.35	
II	E (base scenario)	6.10	6.10	2.6	37.21	
	F	7.25	5.13	2.6	37.21	
	G	6.50	5.72	2.6	37.21	
	H	5.60	6.64	2.6	37.21	

I: Variations of the ceiling height; II: Variations of the width and depth.

- First group (A–D): The floor area of the living room space was equal for all four alternatives; it was 33.35 m2. However, the ceiling height was varied from 2.4 m to 2.8 m. In this study, the ceiling height of 2.4 m was the lowest acceptable height for occupancy and 2.8 m was the maximum height regulated by the building code. The ceiling height of 2.7 m was not considered because it does not align with the exterior design proportions between the window and exterior finish. Therefore, ceiling heights of 2.4 m, 2.5 m, 2.6 m, and 2.8 m were provided using random assignment. In this group, the variation of the ceiling height is the influential factor that affects the future occupants’ spatial perceptions.
- Second group (E–H): The floor shape of the living room space was varied with changes in the width and depth, but the ceiling height remained equal for all alternatives. The floor area for all four scenarios was 37.21 m^2^, but different combinations of widths and depths varying from 5.13 m to 7.25 m were used in order to create the same area. A ceiling height of 2.6 was used in this scenario for all rooms. In this group, the variations of the width and depth are the influential factors that affect the spatial perceptions.

All spaces in the experiment were created virtually using ArchiCAD Version 12 (Graphisoft: Budapest, Hungary). Then, these spaces were exported to the Virtual Building Explorer (VBE) format that provides easy navigation for virtual environments, because this navigation environment is more user friendly [40,41]. The display conditions of the VBE format used the render mode with a headlight, a background mode with the sky, and a 0.98° view cone. The navigation speed and mouse sensitivity were 0.59 s and 22, respectively. A camera was installed at a height of 1.6 m above the floor and it was used for the navigator’s visual fields. All spaces were projected on a 17-inch TFT computer screen in sequence. The screen resolution was 1600 × 900, and the conditions of brightness, color quality, and images did not cause visual problems for navigation.

**Figure 3:**
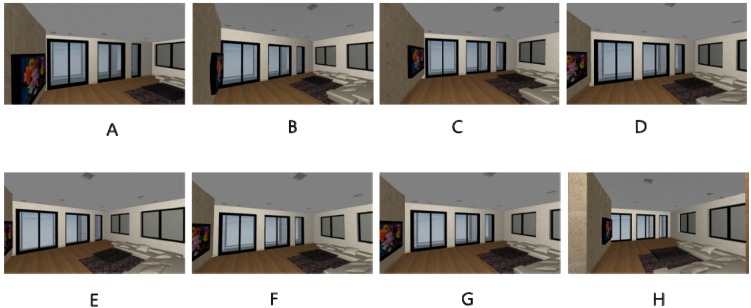
Perspective view of the selected spaces (Ceiling height varied for **A**–**D**, Base scenarios = **A**: 2.4 m; Room shape varied for **E**–**F**, Base scenarios = **G**: 6 m × 6 m).

Perspective views of the eight simulated spaces are illustrated in Figure 3. The floor covering was a light brown wood. The colors of the southern and western walls were light gray; those of the northern and eastern walls were light brown and beige, respectively. The ceiling was a medium gray. A gray-colored five-seat sofa was placed in the southeast area of the room, and the remainder of the space was used for personal use. A coffee table with dimensions of 1.2 m × 0.9 m was placed in front of the sofa. The top area of the table was covered with glass. A light purple rug was placed on the floor underneath the sofa and coffee table. A wall-mounted television was installed on the eastern wall, and the entrance door was in the northern wall.

### 2.2. Participants and Questionnaires

The survey questions used in this study consisted of general questions for participants and self-reported questions for spatial perception. The general questions contained information about the participants such as gender, age, spatial sense of change in space configurations, *etc.* The general questions were used to analyze the demographic characteristics and spatial perception ability of the participants. The health conditions were prescreened in order to satisfy the general standards for participants, e.g., normal vision and the ability to identify colors and variations of spatial configurations in virtual worlds. A seven-point Likert scale was used to answer all questions from strongly disagree (–3 points) to strongly agree (+3 points) as described in Table 2.

**Table 2:** Likert voting scale for questionnaries.

Answer	Scale
Strongly agree	3
Moderately agree	2
Slightly agree	1
Neither agree nor disagree	0
Slightly disagree	−1
Moderately disagree	−2
Strongly disagree	−3

There were thirty-three participants in the experiment based on the living room shapes for SDA. Twenty-three females and ten males who were expecting to buy the house in the future randomly participated in this study. Their age varied from minimal age of 21 to maximal age of 29 years, with a mean age of 24.9 years. All participants were familiar with computer tasks using virtual reality navigation. In the pre-experiments, it was found that non-professionals could not successfully participate in the feedback assignment, because their inexperienced skills in navigating the software interrupted their concentration on the feedback.

The eye height of participants in this study was 1.6 m. The differences of individuals were not considered to collect their responses. Korean Statistical Information Service (http://kosis.kr, 2010) reported that average eye height of 25-year old Korean is 1.62 m for man and 1.49 m for woman, respectively. Also, in the procedure of test in this study, pre-accommodation in the VRE were sufficiently conducted for participants to appropriately control their expectation or bias error according to the height differences.

The self-reported questions for spatial perception used in the survey are presented in Table 3 and Table 4. The organization of the spatial perception questions for the main survey was established logically in order to appropriately interpret the relationship in the time-sequential feedback. The questions provided a time-sequential structure for the correlation between the affordance of the spatial factors and the overall satisfaction in the participants’ spatial perceptions.

**Table 3:** Contents of questionnaire for amenity perception.

Number	Questions Content	Category
A1	How well is your sense of amenity satisfied while walking through this space?	Satisfaction of space
A2	Is the room width conducive to feeling amenity?	spatial size
A3	Is the room depth conducive to feeling amenity?	
A4	Is the room height conducive to feeling amenity?	
A5	Is the room area conducive to feeling amenity?	
A6	Is the ratio of width to depth conducive to feeling amenity?	spatial shape
A7	Is the room shape conducive to feeling amenity?	
A8	Is the ratio of height to area conducive to feeling amenity?	
A9	Is the front window size conducive to feeling amenity?	
A10	Is the right window size conducive to feeling amenity?	
A11	Is the sofa location conducive to feeling amenity?	spatial elements configuration
A12	Is the vacant space size conducive to feeling amenity?	
A13	Is the vacant space location conducive to feeling amenity?	
A14	Is the balcony door location conducive to feeling amenity?	
A15	Is the corridor width to balcony conducive to feeling amenity?	

**Table 4:** Contents of questionnaire for efficiency perception.

Number	Questions Content	Category
E1	How well is your sense of efficiency satisfied while walking through this space?	Satisfaction of space
E2	Is the room width sufficient for serving guests?	spatial size
E3	Is the room depth sufficient for serving guests?	
E4	Is the room height sufficient for serving guests?	
E5	Is the room area sufficient for serving guests?	
E6	Is the ratio of width to depth sufficient for serving guests?	spatial shape
E7	Is the room shape sufficient for serving guests?	
E8	Is the ratio of height to area sufficient for serving guests?	
E9	Is the front window size sufficient for serving guests?	
E10	Is the right window size sufficient for serving guests?	
E11	Is the sofa location sufficient for serving guests?	spatial elements configuration
E12	Is the vacant space sufficient for serving guests?	
E13	Is the vacant space location sufficient for serving guests?	
E14	Is the balcony door location sufficient for serving guests?	
E15	Is the corridor width to balcony sufficient for serving guests?	

The questions regarding the spatial factors were organized referring to the questions in previous POE studies [14,35,53,73,81,85]. Questions were divided into three categories: the effect of the spatial size factors was evaluated using the questions regarding the height, width, and depth; the effect of the spatial shape factors was evaluated using the questions regarding the shape and ratios; and the effect of the spatial element configuration factors was evaluated using the questions regarding the vacant space, furniture location, *etc.* The responses were evaluated at the low level perception (*i.e.*, intervention to the reasoning through activating the emotional experiences in virtual space regarding the previous experience in real spaces).

The questions regarding satisfaction were organized with reference to the previous POE [44,48,86] as well as neuroscience research [46,47,49]. Yasushi argued that the satisfaction of efficiency is affected by the spatial aspects to incerease the ussage; *i.e.*, everyday life activity, utilization of facilites, transproation, and service. Furthermore, the satisfaction of amenity is affected by the spatial aspects to incerease the emotional pleasure; *i.e.*, high quality aesthetic, openness, easiness of community formation, separation from unpleasant facilities, and plenty of nature, *etc.* [29,53].

In this study, efficiency was evaluated regarding the everyday life activity and usage of facilities to support the guest serving activity. Amenity was evaluated regarding aesthetic and openness to the nature to affect emotional pleasure. These responses were evaluated at the high level perception (*i.e.*, reason-based perception through priming the categorical knowledge in order to obtain the expectations regarding satisfaction of the current context).

### 2.3. Occupants’ Participatory Evaluation Procedures and Statistical Analysis Method

A series of experiments were conducted in order to examine the SDA of amenity and efficiency. Each experiment was conducted using the same procedure. In each experiment, the instructions, main tests, and final survey were conducted in a computer laboratory using the following procedure.

- *Step 1: Introduction for 15 min*. Prior to the main tests, instructions were provided about the background of the tests with an explanation of the house plans and the survey procedure that is presented in Figure 2.
- *Step 2: Priming for 15 min*. Two types of framings were conducted in order to elaborate the potential occupants’ perceptions. The framing of categorical knowledge for the terminology of amenity and that for specific behavior in the current design alternatives were conducted. During the priming period, the participants adapted themselves to the current virtual spatial environments where the behavior settings were framed on amenity. The participants were primed to be ready to immediately respond with their judgment using both subconscious and conscious episodic memory activation [37].
- *Step 3: Evaluation of perception (40 min)*. During the survey, the participants navigated eight spaces during the 40-min period and they perceived the spatial conditions of each room. When the participants navigated each room, they concurrently answered the survey questions that corresponded to each spatial element from Number 2 to Number 15 as listed in Table 2. These questions evaluated the perception of each spatial factor that affects the participant’s satisfaction with the amenity.
- *Step 4: Evaluation of overall satisfaction after main survey (5 min)*. After the participants completed the navigation and answers for each room, they answered question Number 1 in Table 2.

In these experiments, the participants were not made aware the changes in the spatial factors before entering the next space, because they should determine the changes in the space independently without prior information. This procedure prevents the “guinea pig effect”. The participants could concentrate on articulating their perceived affordance and satisfaction without bias, because they could maintain the evaluation direction for amenity according to the framing and seamless evaluation during the walk through in the virtual space.

Findings were analyzed using statistics tools. (1) All mean (M) and standard deviation (SD) of responses (A2 and E2–A15 and E15) for the spatial perception in the survey questions in Table 3 were analyzed; (2) A multiple linear regression was performed. The overall satisfaction levels for amenity (A1 and E1) were considered to be dependent variables in the prediction models. All responses (A2–A15) for the spatial perception in the survey questions in Table 3 and Table 4 were used as independent variables. All participant responses of perceptions were included in the initial prediction models as independent variables. Next, the independent variables whose significance levels were lower than 0.05 were selectively included in the final models for the multiple regression analysis; (3) Analysis of variance (ANOVA) tests were used for the models in order to examine whether an acceptable multiple linear relationship existed between the dependent and independent variables.

## 3. Analysis of Occupants’ Perception of Amenity

### 3.1. Participants’ General Characteristics

**Figure 4:**
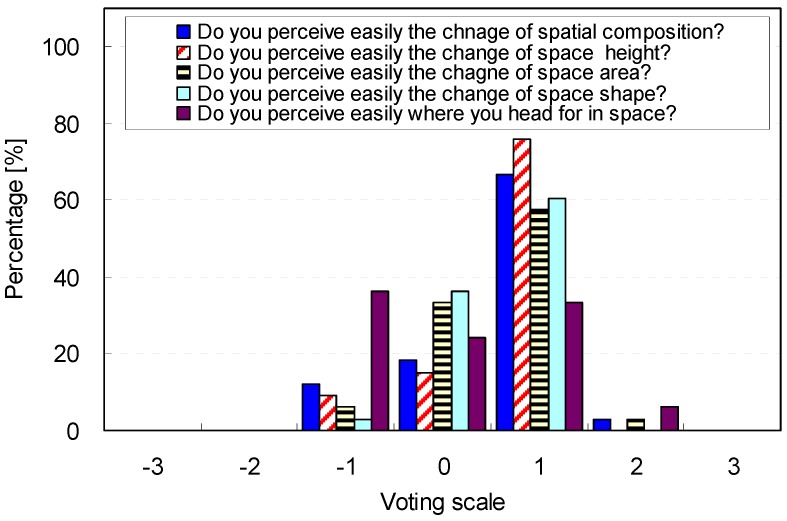
Participants’ ability to perceive change in the spatial components.

The general characteristics of the participants, which can explain their sensitivity to perceiving spatial conditions, are described in Figure 4. Overall, the participants easily perceived changes in the spatial components that influence spatial perceptions. While 69.9% of the participants easily recognized the changes in the spatial components (M = 0.61, SD = 0.71), 12.12% of the participants were not sensitive to these changes.

In more detail, 75.76% of participants answered that they could easily perceive changes in the ceiling height, but 9.09% of participants were not sensitive to these ceiling height changes (M = 0.67, SD = 0.65). The participants also perceived the area changes less sensitively than they perceived the changes of the ceiling height (M = 0.58, SD = 0.66). Furthermore, the participants appropriately perceived changes in the spatial shapes (M = 0.58, SD = 0.56). However, when the participants navigated the spaces, their ability to perceive direction was not excellent (M = 0.09, SD = 0.98). In summary, the distribution of the general characteristics of the participants was not noticeably skewed, although the distribution was not perfectly normal. This indicates that the survey results from the participants were unbiased and that they provide reliable results.

### 3.2. Effect of Spatial Size on the Perception of Amenity

**Figure 5:**
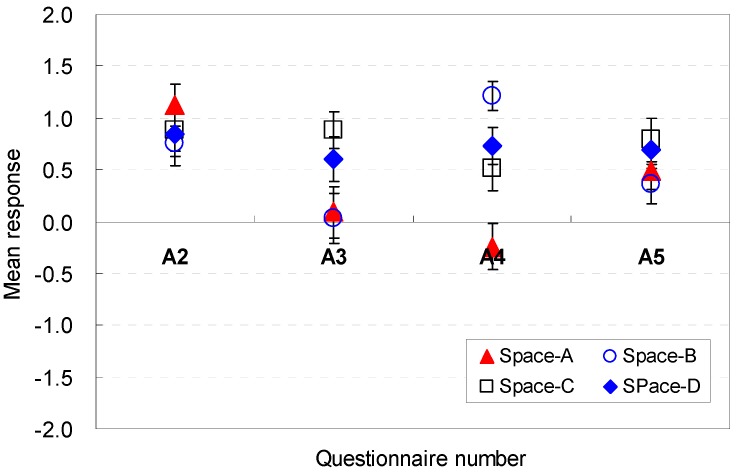
Spatial perception of the spatial size in terms of amenity under height changes.

**Figure 6:**
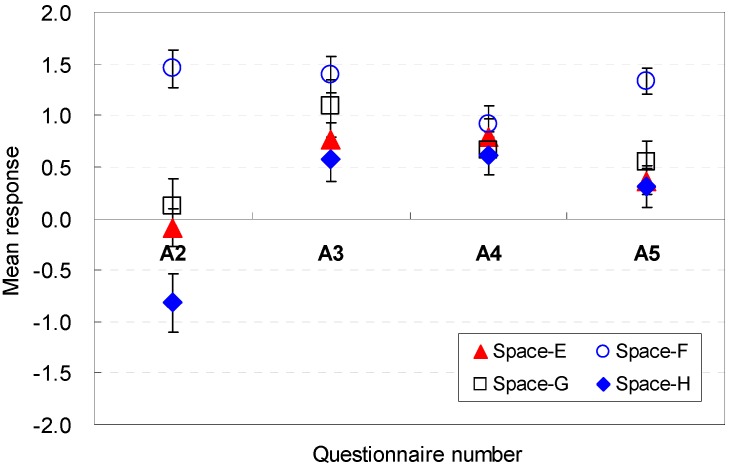
Spatial perception of the spatial size in terms of amenity under floor layout changes.

The occupants’ perceptions of the spatial size factors to conceive the amenity feeling were analyzed. The statistical analysis results for each question are presented in Figure 5 and Figure 6. The width of the space was sufficient to achieve better amenity (A2) for Spaces A, B, C, and D. The ceiling height of 2.4 m (Space A) provided good conditions for better amenity when the floor area was equal for all spaces (M = 1.12, SD = 1.17). The difference in the mean responses was within 0.27 in the Likert scale. These findings indicate that the width was perceived to be wider under lower ceiling height conditions when the height of all rooms remained the same.

However, when the width and depth varied but the height remained the same in Spaces E, F, G, and H, the mean responses exhibited noticeable differences. The most positive responses for amenity were reported for Space F (M = 1.45, SD = 1.06), and the most negative perceptions were reported for Space H (M = −0.82, SD = 1.61). The findings indicate that a wider width is perceived to be a more agreeable condition. 

The depth of the space was sufficient to feel better amenity (A3) for Spaces A, B, C, and D. The change in the depth was an effective element that caused noticeable differences in the mean responses. The lowest depth (Space F) was a strong contributor to the improvement of amenity (M = 1.39, SD = 1.00). The findings indicated that a deeper depth was not an agreeable condition.

The participants exhibited noticeable differences in their perception when the ceiling height varied and the floor area remained equal (A4). Under equal floor shape conditions, the highest ceiling height of 2.8 m in Space B was a strong contributor to the improvement of amenity (M = 1.21, SD = 0.82). However, the ceiling height of 2.4 m for Space A failed to provide excellent perceptions of amenity. These results indicate that the increase in the height was perceived to be an agreeable condition. When the ceiling height was maintained equal and the floor shape was changed, the difference range was within 0.3 in the Likert scale. These results also demonstrated that the participants could clearly articulate their perceived affordances that corresponded to the height variations in the virtual space.

The floor area of the space was also a meaningful factor for the spatial perception in terms of amenity (A5). Compared with the base scenario (Space E), Space F, which had an increased width of 18.85% and a decreased depth of 18.91%, improved the perception of amenity for the space (M = 1.33, SD = 0.74). In contrast, when the floor area remained equal, the increase in the ceiling height deteriorated the spatial perception. However, a slight increase, such as 4.17% (Space C), slightly improved the perception (M = 0.79, SD = 0.22). The results demonstrated that wider widths of the room were perceived to be more agreeable when all areas are the same.

In summary, wider widths are perceived to be more agreeable; deeper depths are not agreeable; ceiling height increases are perceived to be an agreeable condition; and wider widths of the room are perceived as more agreeable when all areas are the same. The occupants’ perception are associated with the *cognitive psychological perspectives and human-environment-interaction theories* as follows: the deeper depths could generate cramped feelings in the participants; the increases in height positively generate feelings of pleasure.

### 3.3. Effect of Spatial Shape on Perception of Amenity

**Figure 7:**
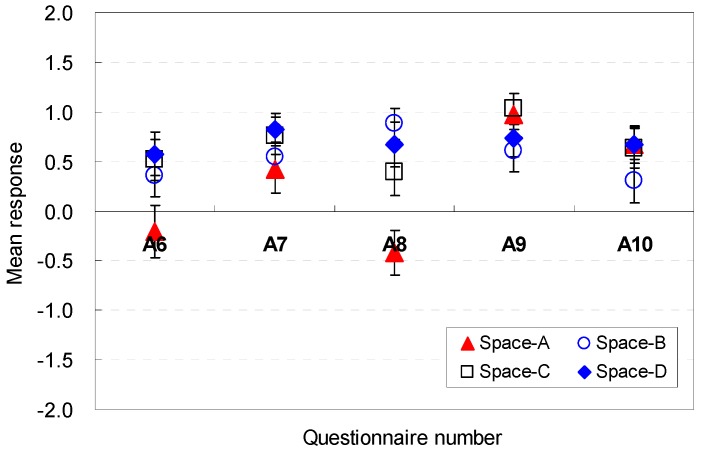
Spatial perception of the spatial shape in terms of amenity under height changes.

**Figure 8:**
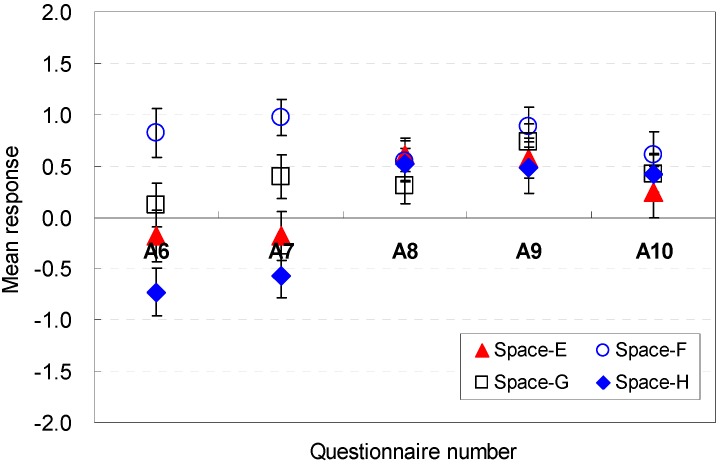
Spatial perception of the spatial shape in terms of amenity under floor layout changes.

The occupants’ perceptions of the spatial configuration factors to conceive the amenity feeling are depicted in Figure 7 and Figure 8. The ratio between the width and depth was a meaningful factor in the perceptions of amenity (A6). When the floor area remained equal, the 2.6 m of ceiling height was evaluated as having better amenity. Changes in the width and depth under equal ceiling height conditions effectively influenced the spatial perceptions. Space F, which was a rectangular shape that had a wider width and a shallower depth, provided the most positive perceptions of amenity (M = 0.82, SD = 1.36). However, the other rectangular shape (Space H), which had a narrower width and a deeper depth, resulted in the most negative perceptions for participants (M = –0.73, SD = 1.33). The data indicate that wider widths in the ratio between the width and depth are an effective factor in maintaining better perceptions of amenity.

The shape of the space was an effective factor in the perceptions of amenity (A7). The rectangular shape (Space F) with a wider width and shallower depth was the most acceptable shape (M = 0.97, SD = 1.02). The other rectangular shape (Space H) generated the most negative spatial perceptions of amenity because it blocked the outdoor views. The findings demonstrated that the rectangular shape of the space with increases in the width was an effective design parameter for improving the perceptions of amenity in living rooms. 

The ratio of ceiling height to area was sufficiently acceptable for enlarging the amenity (A8). When the floor shape remained equal, variations of the ceiling height to area generated noticeable differences in the mean response of amenity. As the ceiling height increased from 2.4 m to 2.8 m, the perception of amenity also increased. Space B, which had the highest ceiling height of 2.8 m, was the most acceptable as an appropriate space for the perception of amenity (M = 0.88, SD = 0.89). However, in Spaces E, F, G, and H, the ratio of the ceiling height to area was not a significant factor in providing clear differences in the perceptions of amenity. The results indicate that the ratio of increasing the ceiling height to the area could contribute to increasing the amenity when the floor area is fixed.

For all spaces, the window dimensions were not critical and did not generate distinguishable differences in the perceptions of amenity (A9, A10). However, the perceptions of amenity were influenced more by changes in the front window conditions than the side window conditions. These results indicate that the front window is a significant factor.

In summary, wider widths in the ratio between the width and depth are effective in maintaining better perceptions of amenity; the rectangular shape of the space with increases in the width is an effective design parameter for improving the perceptions of amenity in living rooms; and the ratio of increasing ceiling height to area is a good contributor to increasing the amenity when the floor area is fixed. The occupants’ perception are associated with the cognitive psychological perspectives and human-environment-interaction theories as follows: the openness provided by the ratio of the wide width effectively generates the feelings of pleasure; the rectangular shape effectively generated open views toward outside; the front window effectively generates the feelings direct access to the outdoor space.

### 3.4. Effect of Spatial Elements Configuration on Perception of Amenity

**Figure 9:**
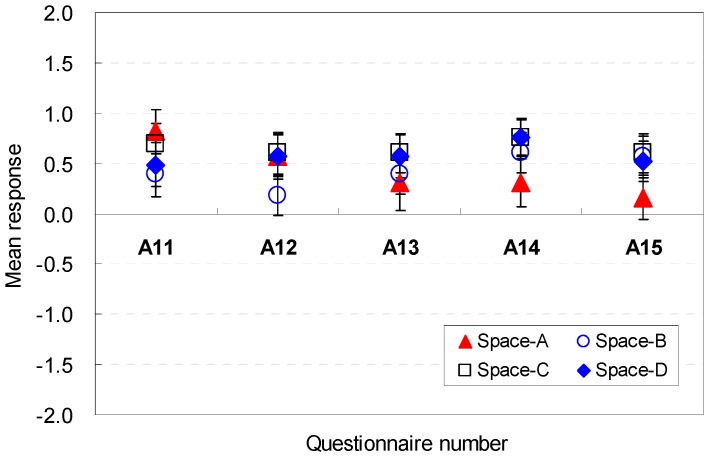
Spatial perception of the spatial configuration in terms of amenity under height changes.

**Figure 10:**
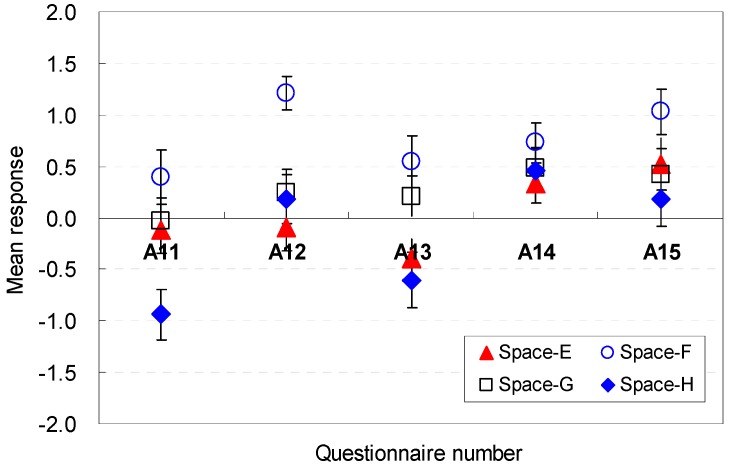
Spatial perception of the spatial configuration in terms of amenity under floor layout changes.

The occupants’ perceptions of the spatial elemental factors to conceive the amenity feeling are analyzed in Figure 9 and Figure 10. For Spaces A, B, C, and D, the participants responded that the position of the sofa was generally acceptable in terms of amenity. For Spaces E, F, G, and H (A11), the furniture layout reduced the spatial perception in terms of amenity. The square shape (Space E), which was the base scenario, generated a mean response close to the neutral point. The findings indicated that the sofa location in this study was agreeable. 

The size of the available space was also sufficiently large for amenity (A12). Space F with a broad width and shallow depth was the most agreeable condition that could afford amenity (M = 1.21, SD = 0.96). The findings demonstrate that the size of the vacant space in spaces with wide widths can more easily afford amenity.

Space F, where the available space was separated from the sofa area and was close to the entrance area, increased the perception of amenity more than the other spaces (A13). These data demonstrate that the available space should be placed without blocking the view to the outside green space by a certain activities. Therefore, Space H, which had a narrow width and deep depth, could not improve the spatial perception of amenity.

The position of the balcony door was an influential factor in the perception of amenity (A14). The participants agreed on the balcony door position. A broad width of the pathway to the balcony was an agreeable condition for the participants’ spatial perception of amenity (A15). Space F, which had a wide width and shallow depth, provided a more agreeable condition to afford amenity. The findings demonstrated that a wider corridor would provide an agreeable condition for amenity.

In summary, the size of the vacant space in a wide width of space was a more agreeable condition to afford amenity; the available space should be placed without blocking the view to the outdoor green space; and a wider corridor provided an agreeable condition for amenity. The occupants’ perception are associated with the cognitive psychological perspectives and human-environment-interaction theories as follows: the participants perceived that the wide areas near the entrance would not disturb the activity and view outdoors, when the participants were sitting and looking toward the outside; the space with a wide width would be perceived to be larger even though all spaces had the same area; a narrow width and deep depth blocked the outdoor view; the wider corridor was perceived to afford more comfortable access to outdoors.

### 3.5. Overall Satisfaction of Space and the Influential Spatial Factors to Affect Satisfaction for Amenity

**Figure 11:**
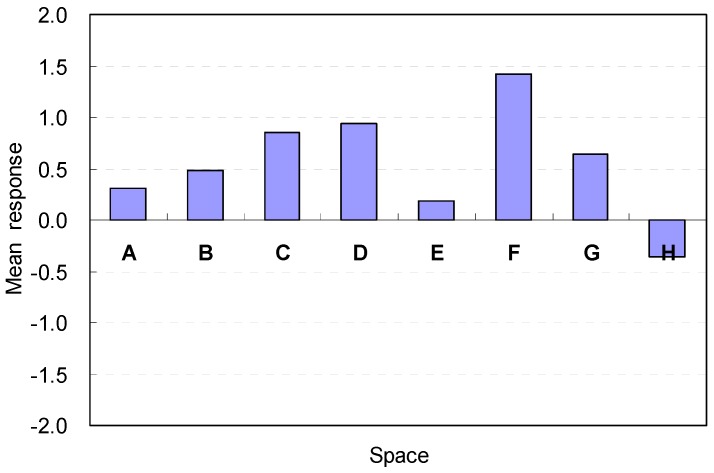
Overall satisfaction of the space in terms of amenity.

The overall satisfaction levels of space in terms of amenity are illustrated in Figure 11. The mean response of the overall satisfaction varied according to the changes in the spatial factors used for the space. Among Spaces A, B, C, and D, Space D with a ceiling height of 2.6 m was the most favorable condition in terms of amenity (A1) (M = 0.94, SD = 0.90). A decrease in the height by 0.1 m to 2.5 m caused a slight decrease in the mean response (M = 0.85, SD = 01.12). The lowest and highest ceiling heights failed to have favorable conditions for the participants. The perceptions of amenity were best when the ceiling height was 2.6 m. However, the perception of the ceiling height of 2.8 m had the most agreeable condition responses during the immediate feedback.

The results indicate that the harmonious composition of the best aesthetic stability and proportion between the ceiling height and width might be acceptable as effective spatial factors in the overall satisfaction of amenity. The immediate perceptions obtained from the environmental perception level based on the participants’ experiences, and the overall satisfaction was obtained through the spatial cognition via recall of spatial experiences.

For Spaces E, F, G, and H, the overall satisfaction in terms of amenity varied significantly corresponding to the space width. Spaces with rectangular floor shapes also generated noticeable satisfaction levels for amenity. The rectangular shape that had the widest width (Space F) was the most favorable spatial condition among all spaces (M = 1.42, SD = 0.90). However, the rectangular shape with the narrowest width and deepest depth (Space H) generated the worst spatial satisfaction in terms of amenity (M = −0.36, SD = 1.34). These results demonstrate that spatial arrangements that provide a clear view to the outdoor area without blocking the path might be an effective spatial factor for amenity.

In summary, the rectangular shape that had the widest width is the most favorable spatial condition in the overall satisfaction of amenity; the overall satisfaction is obtained through the spatial cognition via recall of spatial experiences as well as the immediate perceptions obtained from the environmental perceptions based on the participants’ experiences. Responses for amenity are summarized in Table 5.

**Table 5:** Responses for amenity perception.

Category	Number	Question Content	Findings in Response for Amenity Perception
space	A1	sense of amenity	For Spaces A, B, C, and D, The perceptions of amenity were best when the ceiling height was 2.6 m; the harmonious composition of the best aesthetic stability and proportion between the ceiling height and width might be acceptable as effective spatial factors in the overall satisfaction; For Spaces E, F, G, and H, The rectangular shape that had the widest width (Space F) was the most favorable spatial condition; spatial arrangements that provide a clear view to the outdoor area without blocking the path might be an effective spatial factor for amenity.
spatial size	A2	room width	The most positive responses for amenity were reported for Space F. The most negative perceptions were reported for Space H.
	A3	room depth	The lowest depth (Space F) was a strong contributor to the improvement of amenity
	A4	room height	The highest ceiling height of 2.8 m in Space B was a strong contributor. 2.4 m for Space A failed to provide excellent perceptions
	A5	room area	Space F, improved the perception of amenity for the space. The increase in the ceiling height deteriorated the spatial perception.
spatial shape	A6	ratio of width to depth	Wider widths in the ratio between the width and depth are an effective; Space F provided the most positive perceptions
	A7	room shape	Rectangular shape (Space F) with a wider width and shallower depth was the most acceptable shape
	A8	ratio of height to area	Ratio of increasing the ceiling height to the area could contribute to increasing the amenity; Space B was acceptable
	A9	front window size	Perceptions were influenced more by changes in the front window conditions than the side window conditions
	A10	right window size	Window dimensions were not critical and did not generate distinguishable differences
spatial elements configuration	A11	sofa location	For Spaces A, B, C, and D, was generally acceptable. For Spaces E, F, G, and H (A11), the furniture layout reduced the spatial perception.
	A12	the vacant space size	Space F was the most agreeable condition
	A13	vacant space location	Space F, separated from the sofa area and was close to the entrance area, increased the perception
	A14	balcony door location	A broad width of the pathway to the balcony was an agreeable condition.
	A15	corridor width to balcony	Space F, which had a wide width and shallow depth, provided a more agreeable condition to afford amenity.

The prediction models and ANOVA test results are presented in Table 6. The significance levels for the models were lower than 0.01, which indicates that the models were strongly acceptable. The coefficient of determination (r^2^) for the three models ranged from 0.558 to 0.736, which implies that the reduced error variance for the prediction models ranged from 55.8% to 73.6% when the perceptions of the spatial factors were used to predict the overall satisfaction in terms of amenity. 

**Table 6:** Relationship between spatial factors and occupants’ perception of amenity.

Space	Variable	Unstandardized Coefficient		T	Sig.	ANOVA
		B	Std. Error			
A, B, C, D	(Constant)	0.082	0.091	0.91	0.37	F(5,126) = 31.76; Sig. = 0.00; r^2^ = 0.558
	A2	0.228	0.058	3.92	0.00	
	A3	0.370	0.058	6.34	0.00	
	A4	0.338	0.061	5.50	0.00	
	A10	0.193	0.061	3.18	0.00	
	A15	−0.193	0.066	−2.93	0.00	
E, F, G, H	(Constant)	0.032	0.085	0.38	0.70	F(5,126) = 70.16; Sig. = 0.00; r^2^ = 0.736
	A2	0.312	0.046	6.73	0.00	
	A3	0.234	0.057	4.07	0.00	
	A4	0.171	0.064	2.67	0.01	
	A7	0.284	0.064	4.42	0.00	
	A13	0.133	0.053	2.51	0.01	

variable: question number in Table 3.

For Spaces A, B, C, and D, where the floor shape was the same but the ceiling height was varied, the overall satisfaction level of amenity was influenced by the perceived perception of the width (A2), depth (A3), ceiling height (A4), window size (A10), and width of passage to the balcony (A15). The results indicate that the width, depth, and height of the space may strongly influence the overall satisfaction level in terms of amenity. For Spaces E, F, G, and H, where the floor shape varied but the ceiling height remained the same, the overall satisfaction in terms of amenity was strongly influenced by the perceived perception of the width (A2), depth (A3), ceiling height (A4), space shape (A7), and available space size (A13).

The findings indicate that the spatial shape factors such as width, depth, and ceiling height may significantly influence the overall satisfaction in terms of amenity. These results provide an effective equation for the prediction of the spatial factors conditions when determining the space shape in the participatory design process for living rooms. The spatial factors that form the spatial shape, such as the width, depth, and ceiling height should be considered carefully in order to improve the satisfaction levels for spatial perceptions of amenity.

In summary, the width, depth, and ceiling height are common factors that should be determined carefully in order to improve spatial satisfaction in terms of amenity. More design options, e.g., window size, width of passage to the balcony, space shape, and size of available space, should also be determined carefully in order to improve the occupants’ spatial perceptions of amenity.

## 4. Analysis of Occupants’ Perception of Efficiency

### 4.1. Effect of Spatial Size on Perception of Efficiency

**Figure 12:**
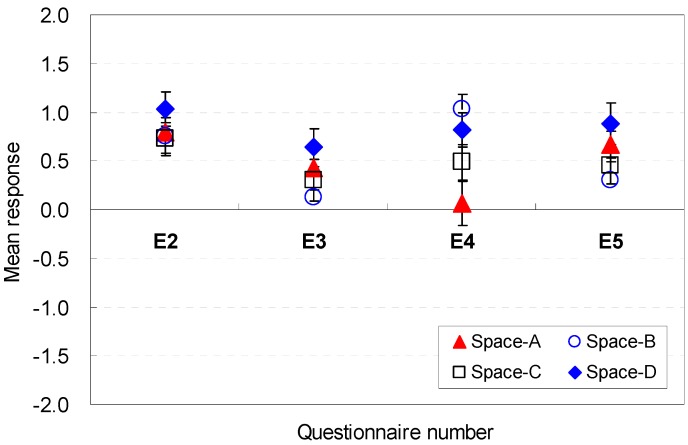
Spatial perception of the spatial size in terms of efficiency under height changes.

**Figure 13:**
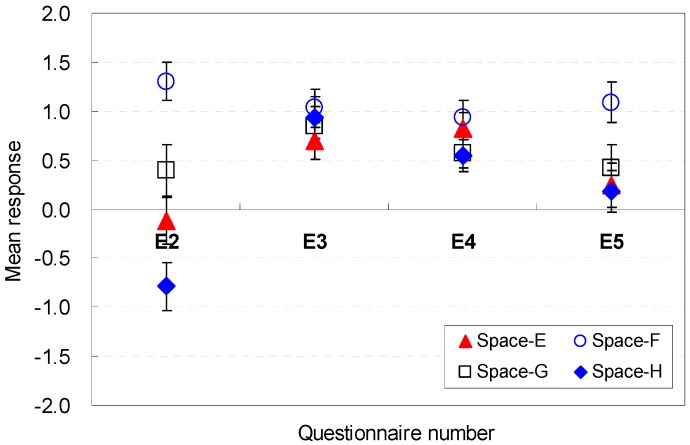
Spatial perception of the spatial size in terms of efficiency under floor layout changes.

The occupants’ perceptions of the spatial size factors to be sufficient for serving guests were analyzed. The statistical analysis results for each question are presented in Figure 12 and Figure 13. The widths were generally acceptable for hosting guests in the Spaces A, B, C, and D (E2). When the ceiling height was 2.6 m (Space D), the perception of efficiency improved slightly (M = 1.03, SD = 0.98) compared with the other space conditions. These findings indicate that the aesthetic stability and proportion between the ceiling height and width of the space might be a meaningful factor for improving perceptions of efficiency under limited ceiling heights.

For Spaces E, F, G, and H, which had significant differences in the mean responses, the difference remained within 2.18 in the Likert scale. Space F, which had the widest width, was the most effective in terms of efficiency for hosting guests in the space (M = 1.39, SD = 1.19). The findings indicated that the wider width is acceptable for hosting guests.

The ceiling height was a good contributor to the perceptions of efficiency (E3) for Spaces A, B, C, and D. The spaces did not have a significant difference in the mean responses. An increase in the width, as in Space F, was an effective contributor to improving perceptions of efficiency (M = 1.07, SD = 0.19). The data indicate that the increase of depth did not significantly improve the perceptions of efficiency.

Noticeable differences of perception were exhibited in participants’ spatial perception for Spaces A, B, C, and D (E4). When the ceiling height was 2.8 m (Space D), the perception of efficiency improved (M = 1.06, SD = 0.18) compared with the other space conditions. The findings indicate that the higher height was sufficiently acceptable for hosting guests. 

The floor area of the space was a meaningful factor for spatial perception in terms of efficiency (E5). Overall, the eight scenarios were acceptable in terms of efficiency for hosting guests in the space. The 2.6 m ceiling height (Space D) was the most effective for improving the spatial perception (M = 0.88, SD = 1.24) in Spaces A, B, C, and D, but the maximum ceiling height of 2.8 m (Space B) caused the worst perception (M = 0.30, SD = 1.07). This result demonstrated that the area was relatively more spacious when the height was lower. 

However, the increase in the width became a strong element for enhancing the perception of efficiency for hosting guests, even though the areas were the same for Spaces E, F, G, and H. Compared with the base scenario (Space E), Space F with a wider width improved the perception of efficiency by 0.85 in the Likert scale. Space F (M = 1.09, SD = 1.16) was the most acceptable condition. This result indicated that the wider width was perceived as acceptable for hosting guests under the same area.

In summary, the wider widths were more acceptable for hosting guests; the increase of depth did not significantly improve efficiency; the higher height was sufficiently acceptable for hosting guests. The occupants’ perception are associated with the *cognitive psychological perspectives and human-environment-interaction theories* as follows: wider width was perceived to provide open views and good accessibility to the outdoors; the long depth was perceived not to provide open views and good accessibility to the outdoors while serving guests; the higher height was perceived to provide open views to the outdoor with participants.

### 4.2. Effect of Spatial Shape on Perception of Efficiency

**Figure 14:**
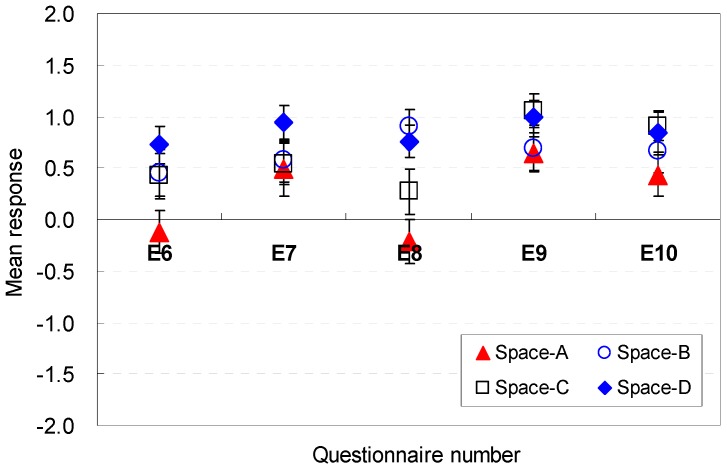
Spatial perception of the spatial shape in terms of efficiency under height changes.

**Figure 15:**
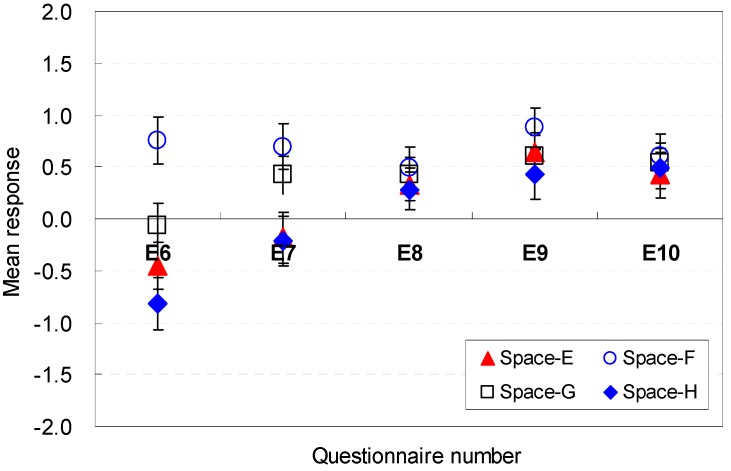
Spatial perception of the spatial shape in terms of efficiency under floor layout changes.

The occupants’ perceptions of the spatial shape factors that were sufficient for serving guests are analyzed in Figure 14 and Figure 15. The ratio between the width and depth was a meaningful factor in the perceptions of efficiency (E6). The height of 2.6 m in Space D (M = 0.73, SD = 1.03) was the most acceptable condition to afford efficiency for serving guests. 

For Spaces E, F, G, and H, the rectangular shape that had a wider width and a shallower depth (Space F) provided positive perceptions of efficiency (M = 1.30, SD = 0.21). This result indicates that spaces with wider widths and shallower depths would be positively effective in serving guests without stress.

The spatial shape was an effective factor in the perceptions of efficiency in all spaces (E7). The rectangular shape (Space F) with a wider width and shallower depth was the more acceptable shape (M = 1.24, SD = 0.18). This result indicates that an increase of the width and a decrease of the depth from a square shape would be effective design parameters for improving the perceptions of efficiency for serving guests without stress in living rooms. 

The ratio of the ceiling height to area was acceptable for enlarging the efficiency (E8). The variation of the ceiling height generated noticeable differences in the mean response of efficiency in Spaces A, B, C, and D. Space B, which had the highest ceiling height of 2.8 m, was evaluated as the appropriate space for enhanced perceptions of efficiency (M = 1.22, SD = 0.16). For Spaces E, F, G, and H, the changes in the width and depth were not significant factors in providing clear differences in the perceptions of efficiency. Furthermore, the results indicate that the same height is not perceived as a meaningful factor to increase the efficiency. 

For all spaces, the window dimensions were not critical and did not generate distinguishable differences in the perceptions of efficiency (E9, E10). The perceptions of efficiency were influenced more by changes in the front window conditions than in the side window conditions. These results indicate that the front window is a significant factor.

In summary, wider widths in the ratio between the width and depth are effective factors in maintaining better perceptions of efficiency; the rectangular shape (Space F) with a wider width and shallower depth was the more acceptable shape; the ratio of the ceiling height to area was acceptable for enlarging the efficiency; the influence of the window size for efficiency is sufficiently weak; the front window is a significant factor. The occupants’ perception are associated with the cognitive psychological perspectives and human-environment-interaction theories as follows: the rectangular shape that had a wider width and a shallower depth is perceived to allow greater accessibility to the outdoors and kitchen without disturbing the serving activities in the living room; the perceptions of efficiency improved as a result of increases in the ceiling height, which expanded the volume of the space; the front window could directly afford access to the outdoor space.

### 4.3. Effect of Spatial Elements Configuration on Perception of Efficiency

**Figure 16:**
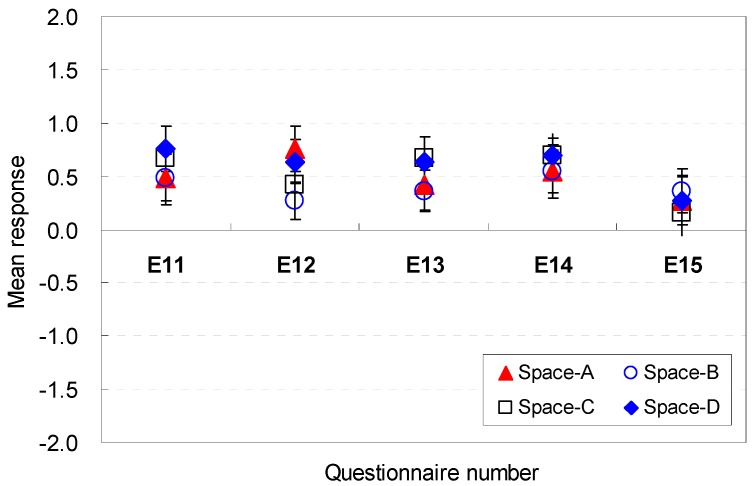
Spatial perception of the spatial configuration in terms of efficiency under height changes.

**Figure 17:**
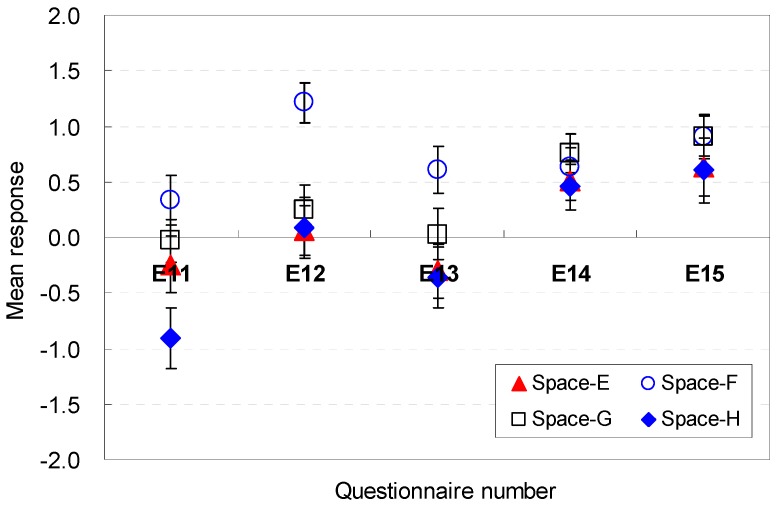
Spatial perception of the spatial configuration in terms of efficiency under floor layout changes.

The occupants’ perceptions of the spatial elemental factors that are sufficient for serving guests are described in Figure 16 and Figure 17. The position of the sofa was generally acceptable in terms of efficiency for Spaces A, B, C, and D (E11). The square shape (Space E), which was the base scenario, generated a mean response close to the neutral point. The findings indicate that the sofa location in this study was agreeable.

The size of the available space was also sufficiently large for efficiency (E12). Space F with a broad width and shallow depth was the most agreeable condition that could afford efficiency (M = 1.02, SD = 0.23). The findings indicate that the size of the vacant space in spaces with wide widths can more easily afford efficiency for serving guests. The findings demonstrate that the space with a wide width was perceived to be larger even though all spaces had the same area. That is, the area near the entrance must remain open for better spatial perception of efficiency.

Space F, where the available space was separated from the sofa area and close to the entrance area, was more acceptable than any other space (E13). These data demonstrate that the available space should be placed without blocking the view to the outside green space during certain activities, e.g., serving guests.

The position of the balcony door was an influential factor in the perception of efficiency (E14). The participants agreed on the balcony door position. A broad width of the pathway to the balcony is an agreeable condition for the participants’ spatial perception of efficiency (E15). Space F, which had a wide width and shallow depth, provided a more agreeable condition to serve guests. This result recommends that the pathway width should be sufficiently wide to go outdoors without disturbing the activities while serving guests in the living room space.

In summary, the available space and sofa area for guests should be separated from the sitting place so that the occupants of the space can move to the balcony area easily without passing through the sofa area; the pathway width should be sufficiently wide to go outdoors without disturbing the activities while serving guests in the living room space. The occupants’ perception are associated with the *cognitive psychological perspectives and human-environment-interaction theories* as follows: the wide areas near the entrance would not disturb the activity for serving guests and viewing outdoors; their views from the sofa were not blocked when they conducted specific activities where the available space was separated from the sofa area and close to the entrance area; sufficient pathway width was guaranteed, and the available space was separated from the sofa area.

### 4.4. Overall Satisfaction of Space and the Influential Spatial Factors to Affect Satisfaction for Efficiency

**Figure 18:**
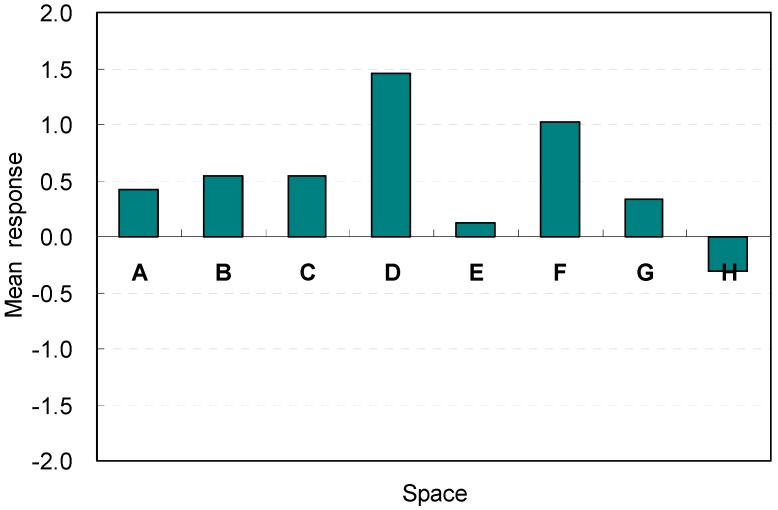
Overall satisfaction of the space in terms of efficiency.

The overall satisfaction levels in terms of efficiency are illustrated in Figure 18. The mean response of the overall satisfaction varied according to the changes in the spatial factors used for the space. For Spaces A, B, C, and D, Space D with a ceiling height of 2.6 m was the most favorable condition in terms of efficiency (E1) (M = 1.45, SD = 3.83). The lowest ceiling height in Space A and the other two heights in Spaces B and C exhibited insignificant differences in their satisfaction levels.

For Spaces E, F, G, and H, the overall satisfaction in terms of efficiency varied significantly according to the space width. Spaces with rectangular floor shapes also generated noticeable satisfaction levels for efficiency (E1). The rectangular shape that had the widest width (Space F) was the best spatial condition to serve guests (M = 1.03, SD = 1.10). However, the rectangular shape with the narrowest width and deepest depth (Space H) failed to provide spatial satisfaction in terms of efficiency (M = –0.30, SD = 1.57). These results demonstrate that spatial arrangements that provide easy accessibility to the outdoor area without interrupting the serving of guests might be an effective spatial factor for efficiency. The flexibility and easy accessibility to serve guests were acceptable as effective spatial factors in the overall satisfaction of efficiency.

Abrupt increases of satisfaction levels occurred in Space D. The perceptions of efficiency were best when the ceiling height was 2.6 m. This response reflects the occupants’ goal-oriented investigation mechanisms: they only responded to the best quality of the spatial conditions for serving guests; moreover, they immediately excluded uncomfortable spatial conditions for serving guests according to their primed behavioral goal (*i.e.*, serving guests).

However, the perception of the ceiling height of 2.8 m had the most agreeable condition responses during the immediate feedback. This occurred because the 2.6 m ceiling height formed the best aesthetic stability for the windows and proportion between the ceiling height and width. The results indicate that the harmonious composition of the best aesthetic stability and proportion between the ceiling height and width might be acceptable as effective spatial factors in the overall satisfaction of efficiency.

In summary, the rectangular shape that had the widest width is the best spatial condition to serve guests; the overall satisfaction is obtained through the spatial cognition via recall of spatial experiences as well as the immediate perceptions obtained from the environmental perceptions based on the participants’ experiences. Responses for efficiency are summarized in Table 7.

The prediction models and ANOVA test results are presented in Table 8. The significance levels for the models were lower than 0.01, which indicates that the models were strongly acceptable. The coefficient of determination (r^2^) for the three models ranged from 0.145 to 0.686, which implies that the reduced error variance for the prediction models ranged from 14.5% to 68.6% when the perceptions of the spatial factors were used to predict the overall satisfaction in terms of efficiency.

**Table 7:** Responses for efficiency perception.

Category	Number	Question Content	Findings in Response for Amenity Perception
space	E1	sense of efficiency	For Spaces A, B, C, and D, Space D with a ceiling height of 2.6 m was the most favorable condition in terms of efficiency. Abrupt increases of satisfaction levels occurred in only Space D. For Spaces E, F, G, and H, The rectangular shape that had the widest width (Space F) was the best spatial condition to serve guests.
spatial size	E2	room width	Space F, which had the widest width, was the most effective in terms of efficiency for hosting guests in the space.
	E3	room depth	All spaces did not have a significant difference; An increase in the width, as in Space F, was an effective contributor
	E4	room height	When the ceiling height was 2.8 m (Space D), the perception of efficiency improved
	E5	room area	Width became a strong element for Spaces E, F, G, and H; 2.6 m ceiling height (Space D) was the most effective, but 2.8 m (Space B) caused the worst perception.
spatial shape	E6	ratio of width to depth	For Spaces E, F, G, and H, the rectangular shape (Space F) provided positive perceptions; 2.6 m height in Space D was the most acceptable condition for serving guests.
	E7	room shape	Increase of the width and a decrease of the depth from a square shape would be effective design parameters for serving guests
	E8	ratio of height to area	Space B, 2.8 m, was evaluated as the appropriate space for enhanced perceptions; the same height is not perceived as a meaningful factor to increase the efficiency.
	E9	front window size	Perceptions were influenced more by changes in the front window conditions than the side window conditions
	E10	right window size	Window dimensions were not critical and did not generate distinguishable differences
spatial elements configuration	E11	sofa location	Sofa location in this study was agreeable; square shape (Space E) generated a mean response close to the neutral point.
	E12	the vacant space size	Space F was the most agreeable condition; the area near the entrance must remain open for better spatial perception
	E13	vacant space location	Space F, separated from the sofa area and was close to the entrance area, increased the perception
	E14	balcony door location	A broad width of the pathway to the balcony was an agreeable condition.
	E15	corridor width to balcony	Space F, which had a wide width and shallow depth, provided a more agreeable condition to serve guests.

**Table 8:** Relationship between spatial factors and occupants’ perception of efficiency.

Space	Variable	Unstandardized Coefficient		T	Sig.	ANOVA
		B	Stander Error			
A, B, C, D	(Constant)	0.211	0.207	1.02	0.31	F(2,129) = 11.10; Sig. = 0.00; r^2^ = 0.145
	E4	0.414	0.164	2.53	0.01	
	E5	0.493	0.172	2.87	0.00	
E, F, G, H	(Constant)	0.093	0.095	0.98	0.33	F(5,126) = 55.02; Sig. = 0.00; r^2^ = 0.686
	E2	0.229	0.064	3.57	0.00	
	E3	0.197	0.064	3.09	0.00	
	E6	0.310	0.081	3.82	0.00	
	E7	0.163	0.077	2.10	0.04	
	E13	0.125	0.063	1.99	0.05	

variable: question number in Table 4.

For Spaces A, B, C, and D, where the floor shape was the same but the ceiling height varied, the relationship between the perceptions of the spatial factors and the overall satisfaction in terms of efficiency was not significant. The results indicate that the variation of the ceiling height may not significantly influence the occupants’ movements to serve the guests in the living room.

For Spaces E, F, G, and H, where the floor shape varied but the ceiling height remained the same, the overall satisfaction in terms of efficiency was strongly influenced by the perceived perception of the width of room (E2), the depth of room (E3), the width-to-depth ratio (E6), the room shape (E7), and vacant space location (E13). In particular, the width-to-depth ratio (E6) was the strongest factor affecting the satisfaction of the space. The results indicate that the core spatial factors such as the width, depth, and ceiling height may significantly influence the overall satisfaction in terms of efficiency. These results provide an effective equation to predict the spatial factor conditions when the space shapes are determined in the participatory design process for living rooms.

In summary, a prediction equation could be used when the design participants determine the conditions of a spatial shape. The width, depth, and ceiling height are common factors that should be determined carefully in order to improve the spatial satisfaction in terms of efficiency; the ratio between the width and depth, and the width may significantly influence the overall satisfaction in terms of efficiency. More design options, e.g., flexibility of the room shape, are available; the depth, space shape, and position of available space should also be determined carefully in order to improve the occupants’ spatial perceptions of efficiency. The findings demonstrate that the relationship between the spatial perception of spatial design factors and occupants’ perceptual satisfaction of efficiency are time-sequential logically presented.

## 5. Discussion

The results of the study demonstrated that a distinct causal relationship between spatial factors and space exists in analyzing the SDA of spatial size and shape. This implication is discussed in order to verify the hypotheses and to signify the aspects of occupants’ perception elaborations while addressing the contribution to practice and theoretical expansion. First, all hypotheses were verified considering the human-environment-interaction theories as follows:

- *Hypothesis group 1 stated that the spatial size factors such as width, depth, and height of space affected the perception of amenity and efficiency.* The previous studies have not clearly described the causal relationship between spatial size factors and space [35,81,82,83,84]. However, the findings of this study demonstrated that high heights and wide widths of room significantly affect the satisfaction of amenity. This result successfully supports the *pleasure-arousal hypothesis*: occupants were most attracted to settings that are moderately arousing and maximally pleasurable for the high height and wide width of the room [52]. In this study, occupants would feel pleasantness in agreeable spatial conditions (the spatial factors of configuration, vacant space, and shape) resulting from agreement with their openness experiences in actual space.
- *Hypothesis group 2 stated that the spatial shape factors such as the width-to-depth ratio, the height-to-area* *ratio, and the room shape affected the perception of amenity and efficiency.* The previous studies have not clearly described the causal relationship between spatial shape factors and space [35,81,82,83,84]. However, the findings of this study demonstrated that a short length and wide width of corridor affected the satisfaction of space in terms of efficiency. This result indicates that the occupants did not feel stress in agreeable spatial conditions (easiness of movement) when serving guests. This result aligns with *social learning theory* [87]: occupants observed, processed, and imitated the behaviors, attitudes, and emotional reactions for serving guests. In this study, occupants perceived a short length of corridor as operant conditioning, because they learned that this condition is desirable [52].
- *Hypothesis group 3* *stated that the overall satisfaction of amenity and efficiency were perceived differently under the same space.* The findings of this study demonstrated that the efficiency satisfaction was influenced by the width-to-depth ratio, the room shape, and vacant space location. In contrast, amenity satisfaction was influenced by the ceiling height. Efficiency was affected by the host movement, and amenity was affected by the pleasure feeling due to openness. The results indicate that amenity and efficiency in the occupants’ perceptions were distinguished in association with the expected situation in order to achieve each occupancy need.

Hypothesis groups 1, 2, and 3 are successfully established. The results imply that the causal relationship between the spatial factors and space reflects the actual experience, with divisions into amenity and efficiency. The causal relationship of the study was limited to the operational interpretation projecting occupants’ time-sequential experiences. Second, the occupants’ perception processing elaborations were observed in association with the *cognitive psychological perspectives and human-environment-interaction theories*, as follows:

- *Occupants’* *perceptions of amenity and efficiency were the highest for the height change, with the highest height. Otherwise, satisfaction perception was the highest for space when the spatial factors were relatively harmonious.* The findings demonstrate that two types perception were observed to compose a complementary two-dimensional path, and low level perceptions (affordance) were directly evaluated instantly, while the satisfaction (a high level perception) was evaluated during the recall the experience.
  In human-environment interaction studies, affordances are regarded as a lower level perception and satisfaction is a high level perception [78,79,80]. In the cognitive psychological mechanism in human-environment-interaction theories, perception, which is the first phase in the overall thought process, involves the interpretation of sensation. Cognition, which is the second phase, is the way that information and knowledge comes to be known, through the actions of perception, reasoning, or intuition [52].
  In this study, the results imply that the possibility of an intentional system could be performed to demonstrate the causal relationship: the correlation can be captured using low-level perception (e.g., perceived affordance) as well as the high-level perception (e.g., attitude, satisfaction); and the correlation between the spatial elements and space can be interpreted reflecting the occupants’ previous time-sequence experiences.
- *Efficiency satisfaction increases abruptly in the evaluation sequence. These response patterns describe that the attention is directly applied to the evaluation of efficiency satisfaction, in which occupants ignored the spatial elements that did not to meet their efficiency needs and in contrast adopted the spatial elements that applied to their efficiency needs.* The findings of this study imply that perception was integrated in association with primed intention with classification between amenity and efficiency. Priming on the occupants’ perceptions would reinforce the evaluation of SDA in comparing their own previous experiences in real spaces with the separation between amenity and efficiency.
  *In the cognitive psychological mechanism in human-environment-interaction theories,* the low-level and high-level perception mechanisms functioned simultaneously because selective attention was provided in the perception mechanism [52]. The perceptions are processed elaborations and amplified when the future occupants were aware of the feelings themselves. In previous studies, this difficulty was addressed in order to capture these perceptions because they are instantly integrated in related brain functions (particularly in the hippocampus) [48,70,71].
  In this study, the results potentially imply that the occupants’ evaluative perception processing elaborations were more activated in the assessment of SDA of amenity and efficiency under the operation conditions such as the primed categorical concepts of amenity and efficiency satisfaction increasing accessibility to long-term memory in association with control of top-down inference strategies and free association increases the activation of evaluative low-level perception in virtual spaces in association with the control of bottom-up perception processing.

Third, the results of this study potentially improve SDA review methods in order to determine the best alternatives in practical use with diminished ambiguities compared with the previous studies. This is exemplified as follows.

- *A series of priming supports to clearly describe the present correlation between spatial factors and space, through comparing occupants’ own experiences of real spaces.* The previous study did not effectively activate the occupants’ perceptions in comparing the alternatives [4,55,62,73,74,75,76,77]. Therefore, the integrated human-environmental perception that describes the current design and previous occupants’ experiences was difficult to capture in the previous conventional POE and VR simulation methods [25,26,32,33,34,35,36]. *In human-environment-interaction theories*, occupants memorized their spatial experience using a time-sequentially causal relationship [80]. Furthermore, the perception of spatial experience is memorized in the long-term memory at the subconscious and conscious level, and it is interpreted with occupants’ own values: individual, social, *etc.*
  However, the results of this study illustrated the distinct result that support for simultaneous integration would be appropriate at low-level and high-level perceptions. The method priming in this study amplified the occupants’ awareness of their feelings and activated the occupants’ perceptions at the level of conscious as well as subconscious in order to evaluate SDA. In this study, the stimuli were compared with the future occupants’ previous experiences and integrated on the occupants’ perceptions. The stimuli were the spatial factors and space built in the VRE.
- *An interpretation of the separating measures that support the description of the relationship as cause and effect: time-sequential causality.* The previous study did not describe the correlation due to reasoning as a manner of separating the measures of occupants’ perceptions. Therefore, the determination cause and effect relationship between the spatial factors and space was difficult to discuss empirically because the occupants could not perceive the analytical structure in the moment [19,42,43,44,45].
  However, the results of this study demonstrated that the distinct relationship was interpreted as a cause and effect relationship: time-sequential causality in human-environmental perception. The method of separating measures would increase the integration of occupants’ perceptions under the activation of occupants’ selective attention; the evaluations were conducted separately: one as immediate assessments of the spatial factors perceptions and the other as satisfaction assessments of the space in recall. In this study, the questions provided the opportunity to statistically analyze the responses using the spatial factors as independent values and satisfaction as the dependent value.
- *The occupant participatory method in this study improves the decision-making process in evaluating SDA regarding the behaviors in the space.* The previous conceptual frameworks lacked a convincing focus on the actors in the housing system, particularly the inhabitants of dwellings, when describing and understanding this set of relationships [18]. The process does not have a coherent series of procedures in the decision-making [74], such as recognition, formulation, designing and screening, choice, deliberating about commitment, action, and feedback. However, the results of this study demonstrated that the decision-making process to select the best space could be improved in order to describe clear SDA as a complementary perception of a two-dimensional path [64,65,66]. This method contributes to solving the difficulty of evaluating SDA regarding the behaviors while considering the potential barriers and opportunities as a participatory process (*i.e.*, designing and screening, choice, and deliberating about commitment), and it contributes to expanding the expectancy-value model and the theory of planned behavior.

## 6. Conclusions

This study examined the best spatial design conditions that satisfy the occupancy needs of amenity and efficiency through analyzing the spatial design adequacy (SDA). In particular, the causal relationship between the space design elements and space were examined under perception processing elaboration in order to activate actual predictive occupancy satisfaction evaluations. The findings of this study are summarized as follows:

1. The spatial size factors demonstrated that wider widths of the space, a rectangular floor shape, and higher ceiling heights were agreeable conditions for amenity. The spatial size factors demonstrated that wider widths of the space were agreeable conditions for hosting guests in terms of efficiency.
2. The spatial shape factors demonstrated that wider widths and shallower depths in the ratio between the width and depth in a rectangular shaped space, and the ratio of increasing height to area were agreeable conditions for amenity. The spatial shape factors demonstrated that wider widths and shallower depths in the ratio between the width and depth in a rectangular shaped space and the ratio of increasing height to area were agreeable conditions for efficiency.
3. The spatial elements configuration demonstrated that the location of the available space without blockages and wider corridors to the balcony access were agreeable conditions for amenity. The spatial elements demonstrated that the location of the available space without blockages and wider corridors to the balcony access were agreeable conditions for serving guests.
4. The overall satisfaction for amenity was determined using the most harmonious design of height rather than the highest height. Furthermore, the rectangular floor shape with the widest width was the best condition for amenity. The width, depth, and height were significantly correlated with the spatial satisfaction in terms of amenity. The window size, width of passage to the balcony, space shape, and size of available space were marginally associated with the spatial satisfaction of amenity.
5. The ratio between the width and depth, and the width may significantly influence the overall satisfaction of efficiency. The window size, width of passage to the balcony, space shape, and size of available space were marginally associated with the spatial satisfaction of efficiency. The rectangular shape that had the widest width was the best spatial condition to serve guests in terms of efficiency. The flexibility of the room shape, depth, space shape, and position of available space should be determined carefully.

The findings of this shows that the causal relationship between the space design elements and space is established under perception processing elaboration for activating the actual predictive occupancy satisfaction evaluation. The results indicate the possibility that the SDA evaluation in the future occupants’ participatory process using VRE, under perception processing elaboration for activating actual predictive occupancy satisfaction evaluation, can be a useful guide to predict the overall satisfaction of amenity and efficiency in the real space. This implies that the expansion of VRE usage benefit in early design review.

When a conflict in SDA arises between design participants, such as designer, contractor, and planner, and potential occupants, it is very important to obtain design consent. The quantification of the visual quality by the future occupants’ is useful in reasonably selecting the best spatial design conditions because a common presentation of the causal relationship between the spatial factors and space could increase the objectivity in selecting the best design among the design stakeholders. Furthermore, this study supports the prediction of the spatial factors that affect the overall satisfaction at a detailed level.

The result of this study implies a convincing explanation to evaluate the expected satisfactory amenity (comfort) and efficiency on SDA in the early design stage. This study evaluates the spatial factors on future occupants’ working memory, whereas POE evaluates occupancy experience on occupants’ retrospective memory. Furthermore, VRE approach has benefit to modify the design because it can be clearly used for architectural design to determine what the influential factors on satisfaction in terms of amenity and efficiency are. This study also suggests the quantitative relationship between the spatial factors and space.

This study was conducted for a limited numbers of spatial factors and space variation that were created virtually. Although the physical environmental factors of living room space influence occupants’ perception, its effectiveness was not examined due to research limitations. Further tests in real surroundings, which examine the influence of physical environmental factors occupants’ perception, would be beneficial for future studies. Since this study investigated the limited numbers of plan layouts in association with the change of depth, width and height of given space conditions, further analysis is recommended under diverse variation of spatial factors. Also, further study would be necessary for space in commercial buildings. Though all participants in this study could not report their perceptions in association with individual eye-height, further evaluation under the conditions reflecting individual eye-height would be beneficial for future studies.

## References

[1] Gibson E., Gebken R. (2003). Design quality in pre-project planning: Applications of the Project Definition Rating Index. Build. Res. Inf..

[2] Nguyen T.H., Shehab T., Gao Z. (2010). Evaluating Sustainability of Architectural Designs Using Building Information Modeling. Open Constr. Build. Technol. J..

[3] Pati D., Park C.-S., Augenbroe G. (2009). Roles of quantified expressions of building performance assessment in facility procurement and management. Build. Environ..

[4] Cole R.J. (2005). Building environmental assessment methods: Redefining intentions and roles. Build. Res. Inf..

[5] Al-Kodmany K. (2001). Visualization tools and methods for participatory planning and design. J. Urban Technol..

[6] Shin D.-H., Shin Y.-J. (2011). Consumers’ Trust in Virtual Mall Shopping: The Role of Social Presence and Perceived Security. Int. J. Hum. Comput. Interact..

[7] Harun S.N., Hamid M.Y., Talib A., Rahim Z.A. (2011). “Usability Evaluation”: Criteria for Quality Architecture In-Use. Procedia Eng..

[8] Zeiler W., Savanovic P. (2009). Integral Morphological C-K Design Approach for Multidisciplinary Building Design. Archit. Eng. Des. Manag..

[9] Lai A.C.K., Mui K.W., Wong L.T., Law L.Y. (2009). An evaluation model for indoor environmental quality (IEQ) acceptance in residential buildings. Energy Build..

[10] Lai J.H.K., Yik F.W.H. (2009). Perception of importance and performance of the indoor environmental quality of high-rise residential buildings. Build. Environ..

[11] Kamaruzzaman S.N., Egbu C.O., Zawawi E.M.A., Ali A.S., Che-Ani A.I. (2011). The effect of indoor environmental quality on occupants’ perception of performance: A case study of refurbished historic buildings in Malaysia. Energy Build..

[12] Kim S.-M., Lee J.-H., Moon H.J., Kim S. (2012). Improvement of Indoor Living Environment by Occupants’ Preferences for Heat Recovery Ventilators in High-Rise Residential Buildings. Indoor Built Environ..

[13] Monfared I.G., Sharples S. (2011). Occupants’ perceptions and expectations of a green office building: A longitudinal case study. Archit. Sci. Rev..

[14] Bonaiuto M., Fornara F., Bonnes M. (2003). Indexes of perceived residential environment quality and neighbourhood attachment in urban environments: A confirmation study on the city of Rome. Landsc. Urban Plan..

[15] Whyte J., Gann D., Whyte J.K., Gann D.M. (2003). Design Quality Indicators: Work in progress. Build. Res. Inf..

[16] Frontczak M., Schiavon S., Goins J., Arens E., Zhang H., Wargocki P. (2012). Quantitative relationships between occupant satisfaction and satisfaction aspects of indoor environmental quality and building design. Indoor Air.

[17] Peretti C. Indoor Environmental Quality Surveys: A Brief Literature Review. http://escholarship.org/uc/item/0wb1v0ss#.

[18] Coolen H.C.C.H. The relevance of the concepts of affordance and behavior setting for housing research. Proceedings of the ENHR 2014 Beyond Globalisation. Remaking House Policy a Complex World.

[19] Stevenson F., Leaman A. (2010). Evaluating housing performance in relation to human behaviour: New challenges. Build. Res. Inf..

[20] Taylor P. (2006). Post-Occupancy Indoor Environmental Post-Occupancy Indoor Environmental Quality Evaluation of Student Housing Facilities. Archit. Eng. Des. Manag..

[21] Cho S., Lee T., Kim J. (2011). Residents’ satisfaction of indoor environmental quality in their old apartment homes IBE 2010. Indoor Built Environ..

[22] Korea Consumer Agency (2005). Analysis of Damage Cases in Apartment.

[23] Hisako K., ASAMI Y. (2003). Evaluation of Amenity (translation in korean). Evaluation of Residential Environment. Evaluation of Living Environment.

[24] Humiko I., ASAMI Y. (2003). Evaluation of Efficiency. Evaluation of Residential Environment. Evaluation of Living Environment.

[25] Zimmerman A., Martin M. (2001). Post-occupancy evaluation: Benefits and barriers. Build. Res. Inf..

[26] Agha-Hossein M.M., El-Jouzi S., Elmualim A.A., Ellis J., Williams M. (2013). Post-occupancy studies of an office environment: Energy performance and occupants’ satisfaction. Build. Environ..

[27] GhaffarianHoseini A., Dahlan N.D., Berardi U., GhaffarianHoseini A., Makaremi N., GhaffarianHoseini M. (2013). Sustainable energy performances of green buildings: A review of current theories, implementations and challenges. Renew. Sustain. Energy Rev..

[28] Zuo J., Zhao Z.-Y. (2014). Green building research–current status and future agenda: A review. Renew. Sustain. Energy Rev..

[29] Kim M.J., Oh M.W., Kim J.T. (2013). A method for evaluating the performance of green buildings with a focus on user experience. Energy Build..

[30] Chau C.K., Tse M.S., Chung K.Y. (2010). A choice experiment to estimate the effect of green experience on preferences and willingness-to-pay for green building attributes. Build. Environ..

[31] Paul W.L., Taylor P.A. (2008). A comparison of occupant comfort and satisfaction between a green building and a conventional building. Build. Environ..

[32] Kassem M., Iqbal N., Kelly G., Lockley S., Dawood N. (2014). Building Information Modelling: Collaborative Design Process Protocols. J. Inf. Technol. Constr..

[33] Shen W., Shen Q., Sun Q. (2012). Building Information Modeling-based user activity simulation and evaluation method for improving designer–user communications. Autom. Constr..

[34] Kumar S., Hedrick M., Wiacek C., Messner J.I. (2011). Developing an experienced-based design review application for healthcare facilities using a 3D game engine. J. Inf. Technol. Constr..

[35] Frontczak M., Wargocki P. (2011). Literature survey on how different factors influence human comfort in indoor environments. Build. Environ..

[36] Markus T.A. (2003). Lessons from the Design Quality Indicator. Build. Res.Inf..

[37] Luo Z., Luo W., Chen I.-M.I., Jiao R.J., Duh H.B. (2010). Spatial Representation of a Virtual Room Space: Perspective and Vertical Movement. Int. J. Hum. Comput. Interact..

[38] Hartmann T., Fischer M. (2007). Supporting the constructability review with 3D/4D models. Build. Res. Inf..

[39] Franz G., von der Heyde M., Bülthoff H.H. (2005). An empirical approach to the experience of architectural space in virtual reality—Exploring relations between features and affective appraisals of rectangular indoor spaces. Autom. Constr..

[40] Arayici Y., Coates P., Koskela L., Kagioglou M., Usher C., O’Reilly K. (2011). Technology adoption in the BIM implementation for lean architectural practice. Autom. Constr..

[41] Pauwels P., de Meyer R., van Campenhout J. Visualisation of semantic architectural information within a game engine environment. Proceedings of the 10th International Conference on C onstruction Applications of Virtual Reality.

[42] Bordass B., Leaman A. (2005). Making feedback and post-occupancy evaluation routine 3: Case studies of the use of techniques in the feedback portfolio. Build. Res. Inf..

[43] Kwon S.-H., Chun C., Kwak R.-Y. (2011). Relationship between quality of building maintenance management services for indoor environmental quality and occupant satisfaction. Build. Environ..

[44] Lee Y.S., Guerin D.A. (2009). Indoor Environmental Quality Related to Occupant Satisfaction and Performance in LEED-certified Buildings. Indoor Built Environ..

[45] Humphreys M.A. (2005). Quantifying occupant comfort: Are combined indices of the indoor environment practicable?. Build. Res. Inf..

[46] Kelly J.W., McNamara T.P. (2010). Reference frames during the acquisition and development of spatial memories. Cognition.

[47] Conway M.A. (2009). Episodic memories. Neuropsychologia.

[48] Moscovitch M., Nadel L., Winocur G., Gilboa A., Rosenbaum R.S. (2006). The cognitive neuroscience of remote episodic, semantic and spatial memory. Curr. Opin. Neurobiol..

[49] Burgess N., Maguire E.A., O’Keefe J. (2002). The Human Hippocampus and Spatial and Episodic Memory. Neuron.

[50] Ozok A.A., Komlodi A. (2009). Better in 3D? An Empirical Investigation of User Satisfaction and Preferences Concerning Two-Dimensional and Three-Dimensional Product Representations in Business-to-Consumer E-Commerce. Int. J. Hum. Comput. Interact..

[51] Nash E.B., Edwards G.W., Thompson J.A., Barfield W. (2000). A Review of Presence and Performance in Virtual Environments. Int. J. Hum. Comput. Interact..

[52] Kopec D. (2012). Environmental Psychology for Design.

[53] Yasushi A. (2003). Evaluation of Residential Environment.

[54] Dewulf G., van Meel J. (2004). Sense and nonsense of measuring design quality. Build. Res. Inf..

[55] Eley J. (2004). Design quality in buildings. Build. Res. Inf..

[56] Land and housing institute A Research on Residential Conditions 2012. www.korea.kr/common/download.do?tblKey=EDN&fileId=208315.

[57] Kang N.N., Lee T.K., Kim J.T. (2012). Characteristics of the Quality of Korean High-Rise Apartments Using the Health Performance Indicator. Indoor Built Environ..

[58] Malkoc E., Ozkan M.B. (2010). Post-occupancy Evaluation of a Built Environment: The Case of Konak Square (Izmir, Turkey). Indoor Built Environ..

[59] Cole-Colander C. (2003). Designing the Customer Experience. Build. Res. Inf..

[60] Yildirim K., Capanoglu A., Cagatay K. (2011). The Effects of Physical Environmental Factors on Students’ Perceptions in Computer Classrooms. Indoor Built Environ..

[61] Andrews C.J., Senick J.A., Wener R.E., Mallory-Hill S., Preiser W.F.E., Watson C. (2012). Incorporating Occupant Perceptions and Behaviro into BIM. Enhancing Builing Performance.

[62] Conniff A., Craig T., Laing R., Galán-Díaz C. (2010). A comparison of active navigation and passive observation of desktop models of future built environments. Des. Stud..

[63] Ernst M.O., Lange C., Newell F.N. (2007). Multisensory recognition of actively explored objects. Can. J. Exp. Psychol..

[64] Bechara A., Damasio H., Damasio A.R., Lee G.P. (1999). Different contributions of the human amygdala and ventromedial prefrontal cortex to decision-making. J. Neurosci..

[65] Bechara A. (2000). Emotion, Decision Making and the Orbitofrontal Cortex. Cereb. Cortex.

[66] Rahman S., Sahakian B.J., Cardinal R.N., Rogers R.D., Robbins T.W. (2001). Decision making and neuropsychiatry. Trends Cogn. Sci..

[67] Bogacz R. (2007). Optimal decision-making theories: Linking neurobiology with behaviour. Trends Cogn. Sci..

[68] Smith P.L., Ratcliff R. (2004). Psychology and neurobiology of simple decisions. Trends Neurosci..

[69] Körding K.P., Beierholm U., Ma W.J., Quartz S., Tenenbaum J.B., Shams L. (2007). Causal inference in multisensory perception. PLoS ONE.

[70] Pals R., Steg L., Dontje J., Siero F.W., van der Zee K.I. (2014). Physical features, coherence and positive outcomes of person-environment interactions: A virtual reality study. J. Environ. Psychol..

[71] Hartson R. (2003). Cognitive, physical, sensory, and functional affordances in interaction design. Behav. Inf. Technol..

[72] Dennett D.C. (1983). Intentional systems in cognitive ethology: The “Panglossian paradigm” defended. Behav. Brain Sci..

[73] Lee Y.-J. (2008). Subjective quality of life measurement in Taipei. Build. Environ..

[74] Jansen S.J.T., Coolen H.C.C.H., Goetgeluk R.W. (2011). The Measurement and Analysis of Housing Preference and Choice.

[75] Prasad S. (2004). Clarifying intentions: The design quality indicator. Build. Res. Inf..

[76] Shen W. Management and Innovation for a Sustainable Built Environment.

[77] Koleva B., Anastasi R.O.B., Greenhalgh C., Rodden T.O.M., Green J., Ghali A., Pridmore T., Gaver B., Boucher A., Walker B. (2005). Expected, Sensed, and Desired: A Framework for Designing Sensing-Based Interaction. ACM Trans. Comput. Interatcion.

[78] Lee K.-I., Yeom D.-W. (2011). Comparative study for satisfaction level of green apartment residents. Build. Environ..

[79] Ilesanmi A.O. (2010). Post-occupancy evaluation and residents’ satisfaction with public housing in Lagos, Nigeria. J. Build. Apprais..

[80] Abbaszadeh S., Zagreus L., Lehrer D., Huizenga C. (2006). Occupant Satisfaction with Indoor Environmental Quality in Green Buildings. Proc. Healthy Build..

[81] Frontczak M., Andersen R.V., Wargocki P. (2012). Questionnaire survey on factors influencing comfort with indoor environmental quality in Danish housing. Build. Environ..

[82] Kim J., de Dear R. (2012). Nonlinear relationships between individual IEQ factors and overall workspace satisfaction. Build. Environ..

[83] Minami K. (2007). A Post-Occupancy Evaluation of Layout Changes Made to KEP Adaptable Housing. J. Asian Archit. Build. Eng..

[84] Fang Y. (2006). Residential Satisfaction, Moving Intention and Moving Behaviours: A Study of Redeveloped Neighbourhoods in Inner-City Beijing. Hous. Stud..

[85] Gzsoy A., Problems E. (1998). Spatial Adaptability and Flexibility as Parameters of User Satisfaction for Quality Housing. Build. Environ..

[86] Bassanino M., Fernando T., Wu K.-C. (2013). Can virtual workspaces enhance team communication and collaboration in design review meetings?. Archit. Eng. Des. Manag..

[87] Gosling S.D., Ko S.J., Mannarelli T., Morris M.E. (2003). A room with a cue: Personality judgments based on offices and bedrooms. J. Personal. Soc. Psychol..

